# A Stepwise Technique for Retrieval of a Seized and Stripped Dental Implant Cover Screw: A Case Report

**DOI:** 10.3390/reports9030304

**Published:** 2026-09-10

**Authors:** Keisuke Seki, Kenji Ito, Juntaro Sanada, Taiki Akiyama, Yoshiyuki Hagiwara

**Affiliations:** 1 Implant Dentistry, Nihon University School of Dentistry Dental Hospital, Tokyo 101-8310, Japan; 2 Department of Partial Denture Prosthodontics, Nihon University School of Dentistry, Tokyo 101-8310, Japan; 3 Department of Comprehensive Dentistry and Clinical Education, Nihon University School of Dentistry, Tokyo 101-8310, Japan

**Keywords:** cold welding, cover screw, crown remover, dental implant, stripped screw

## Abstract

**Background and Clinical Significance:** In two-stage implant treatment the cover screw must be removed at second-stage surgery. Removal is occasionally prevented by cold welding or seizure. When the drive slot is damaged by escalating torque, no standard salvage protocol has been established, and the implant body itself may ultimately require removal. **Case Presentation:** During second-stage surgery for three implants placed in the mandibular right posterior region (#44, #45, #46) of a 68-year-old man, the cover screw at the #46 site was seized and could not be loosened with a manual screwdriver. An attempt at removal with a contra-angle implant driver, set first to 20 Ncm and then to 35 Ncm, stripped the drive slot. The procedure was suspended, and a revision was performed two weeks later. A tungsten carbide bur was used under water irrigation to prepare a straight slot across the damaged screw head, and a crown remover with a narrowed flat-blade tip was engaged in this slot. The screw was retrieved intact and the internal connection of the implant remained serviceable. A healing abutment was connected and the implant was restored with a definitive prosthesis, which was in function at the most recent review. At seven weeks after delivery, the distance from the implant platform to the crestal bone was greater at #46 than at the two adjacent implants. **Conclusions:** This report describes a salvage technique that preserved the implant body without the use of a system-specific retrieval kit. The peri-implant bone level observed at the treated site indicates that the procedure may have been accompanied by a loss of crestal bone, and whether the technique is reproducible will require further cases.

## 1. Introduction and Clinical Significance

Dental implants have become a globally recognized and reliable restorative treatment for tooth loss [1]. Surgical placement typically follows either a one-stage or a two-stage protocol. In the two-stage approach, the implant body is submerged and protected by a cover screw during the initial surgery, allowing the mucosa to be closed for submerged healing. After a sufficient healing period to ensure osseointegration, a second-stage surgery is performed to remove the cover screw and connect a healing abutment [2]. The primary advantage of this method is the complete isolation of the implant from the oral environment, which minimizes infection risk and facilitates stable healing, particularly in cases involving simultaneous bone augmentation [3]. Consequently, the two-stage procedure is often the preferred choice for patients with poor bone quality or compromised healing capacity.

Commonly reported prosthetic complications include screw loosening or fracture, abutment damage, and prosthesis detachment, all of which present challenges for long-term maintenance [4,5]. While most complications occur after the final prosthesis is delivered, difficulties in removing cover screws can arise as early as the second-stage surgery. Three mechanisms should be distinguished. Cold welding is the adhesion of two metal surfaces at the atomic level, which occurs when the passive oxide layer between them is disrupted and direct metal-to-metal contact is established [6,7]. Seizure is the resulting state in which the two components can no longer be separated by the torque normally required. Bone overgrowth is a separate situation in which newly formed bone extends over the head or the margin of the cover screw and mechanically obstructs its removal. In the present case no bone was found over the screw, so the first two mechanisms were considered.

Cover screw complications have been reported to occur in 0.5–8% of cases, and this figure may be higher where a conical connection design is used [5]. A small number of reports have addressed the management of a seized cover screw. Bhuvaneswarri and Chandrasekaran described the retrieval of an adhered cover screw [8], and Óvári et al. reported a cold-welded cover screw whose drive slot became worn during attempted removal; that screw was retrieved by drilling through its centre with a tapered carbide bur supplied in a manufacturer-specific retrieval kit [5]. Those authors cautioned, however, that the method may damage the internal threads where bone has grown into the implant, and recommended it only where that risk is absent. A summary of reported retrieval techniques is presented in Table 1.

No standardised protocol exists, and clinicians are often left to manage these situations without dedicated instruments. In the worst case, improper management can lead to the loss of the entire implant body. Therefore, although the complication involves only a cover screw, it can substantially impair the patient’s oral quality of life, in a manner comparable to peri-implantitis.

In the present report we describe a seized cover screw whose drive slot had been stripped during attempted removal. A new slot was prepared on the external surface of the screw head with a carbide bur, and the screw was unscrewed intact using a modified crown remover. In contrast to previously described approaches, the technique required neither a system-specific retrieval kit nor instrumentation within the internal channel of the implant. We report the procedure, its outcome, and the peri-implant bone level observed at the treated site.

## 2. Case Presentation

This case report was prepared in accordance with the CARE Guidelines [11].

### 2.1. Patient Background and Chief Complaint

In September 2025 a 68-year-old man presented to our clinic for implant treatment to restore missing teeth in the mandibular right posterior region (#44, #45 and #46). His medical history included hypertension, diagnosed five years earlier and treated with an antihypertensive agent, and hyperuricemia, treated with a xanthine oxidase inhibitor. Behçet’s disease had been diagnosed at the age of 20, with oral aphthous ulceration and febrile episodes; he had received oral prednisolone from the age of 20 to 25 and had been free of symptoms and of medication for this condition for the past ten years. Colorectal cancer had been resected at the age of 59, without radiotherapy or chemotherapy, and he remained under observation with no medication. He had never received a bisphosphonate or denosumab. He lost his sight following a retinal haemorrhage at the age of 20, attributed to Behçet’s disease, and is now totally blind. He attends appointments with a guide helper. His oral hygiene was assessed as suboptimal, although he brushed regularly. He also reported a gradual decline in renal function, for which no laboratory data were available for review. Preoperative imaging showed a diffuse increase in the radiopacity of the mandible and visually homogeneous, radiopaque cortical bone bilaterally. At the contralateral #36 site, implant placement had previously been complicated by extreme bone density, which led to failure of osseointegration and required reimplantation.

### 2.2. Implant Placement (Stage-One Surgery)

Three implant bodies (Straumann Bone Level; Institut Straumann AG, Basel, Switzerland) were placed according to the manufacturer’s protocol: 3.3 × 8 mm with a Narrow CrossFit (NC) connection at #44 and #45, and 4.1 × 8 mm with a Regular CrossFit (RC) connection at #46. According to the manufacturer’s material data, the implant bodies were made of Roxolid, a titanium–zirconium alloy containing 15 ± 2% zirconium with titanium as the balance (minimum tensile strength 850 MPa; modulus of elasticity 98 GPa). The cover screws were made of commercially pure titanium grade 4 [12] and were new, single-use components supplied by the manufacturer. The bone quality was classified as Type 1 according to the Lekholm and Zarb classification [13], and excellent primary stability was achieved for all implants. The cover screws were hand-tightened using a manual screwdriver (SCS Screwdriver; Straumann) without the use of a torque wrench, after which the full-thickness flaps were repositioned and sutured for submerged healing.

### 2.3. Second-Stage Surgery: Complication in Cover Screw Removal

After a submerged healing period of approximately four months, second-stage surgery was performed for healing abutment connection. Under local anesthesia (Epilido; Nipro Corp., Osaka, Japan), a crestal incision was made and a full-thickness flap was reflected. While the cover screws at sites #44 and #45 were easily removed using the manual screwdriver, the cover screw at site #46 remained immobile.

**Figure 1:**
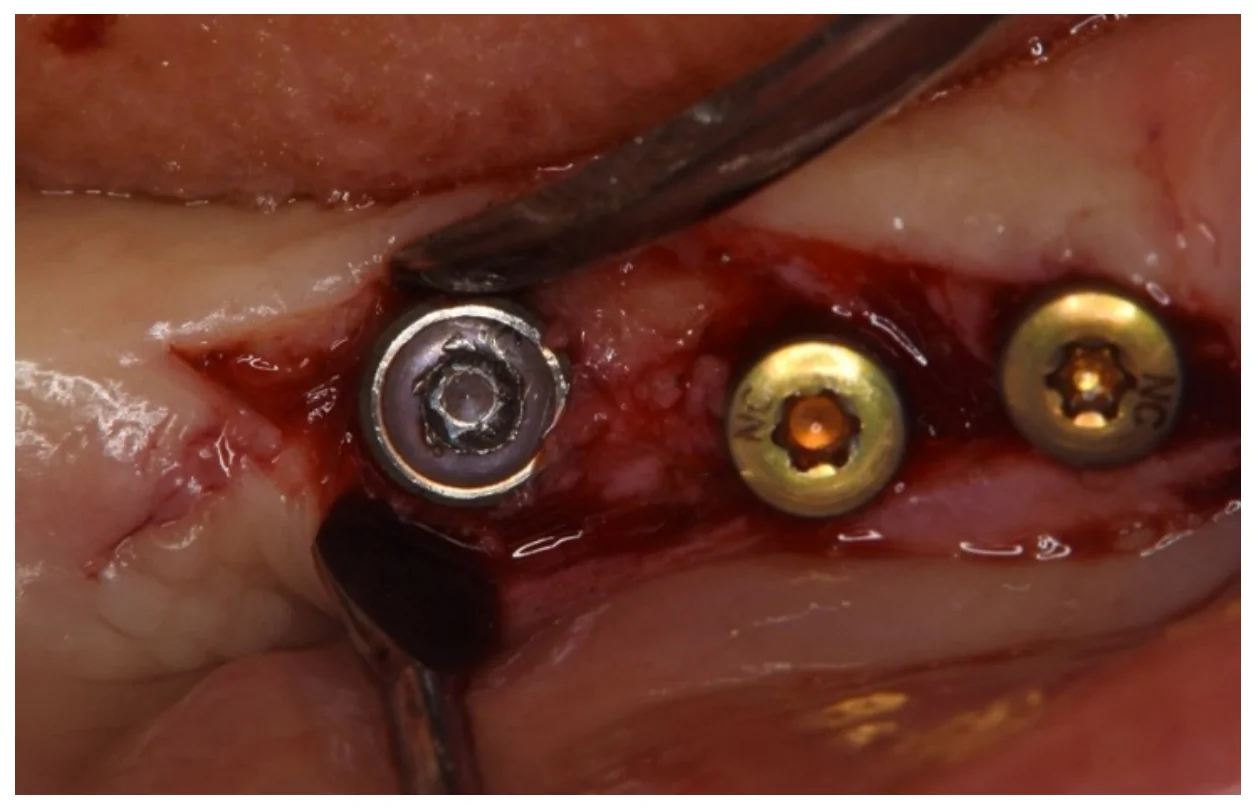
Intraoperative intraoral photograph taken during the second-stage surgery. Occlusal view showing the stripped drive slot of the #46 cover screw. The original SCS configuration can be seen at #44 and #45.

A contra-angle implant driver (NSK; Nakanishi Inc., Tochigi, Japan) was applied at 20 Ncm, but the screw did not loosen. The driver was used with torque attachments of 20 Ncm and 35 Ncm, and no intermediate value was available. When the 35 Ncm attachment was used, the internal drive slot of the screw was stripped, rendering manual removal impossible (Figure 1). No clinical evidence of bone overgrowth or soft tissue interference was observed. To avoid further risk of internal thread damage to the implant body, the site was thoroughly irrigated and closed with simple interrupted sutures (5-0 Monocryl; Ethicon, NJ, USA).

### 2.4. Revision Surgery: Removal of the Seized Cover Screw

**Figure 2:**
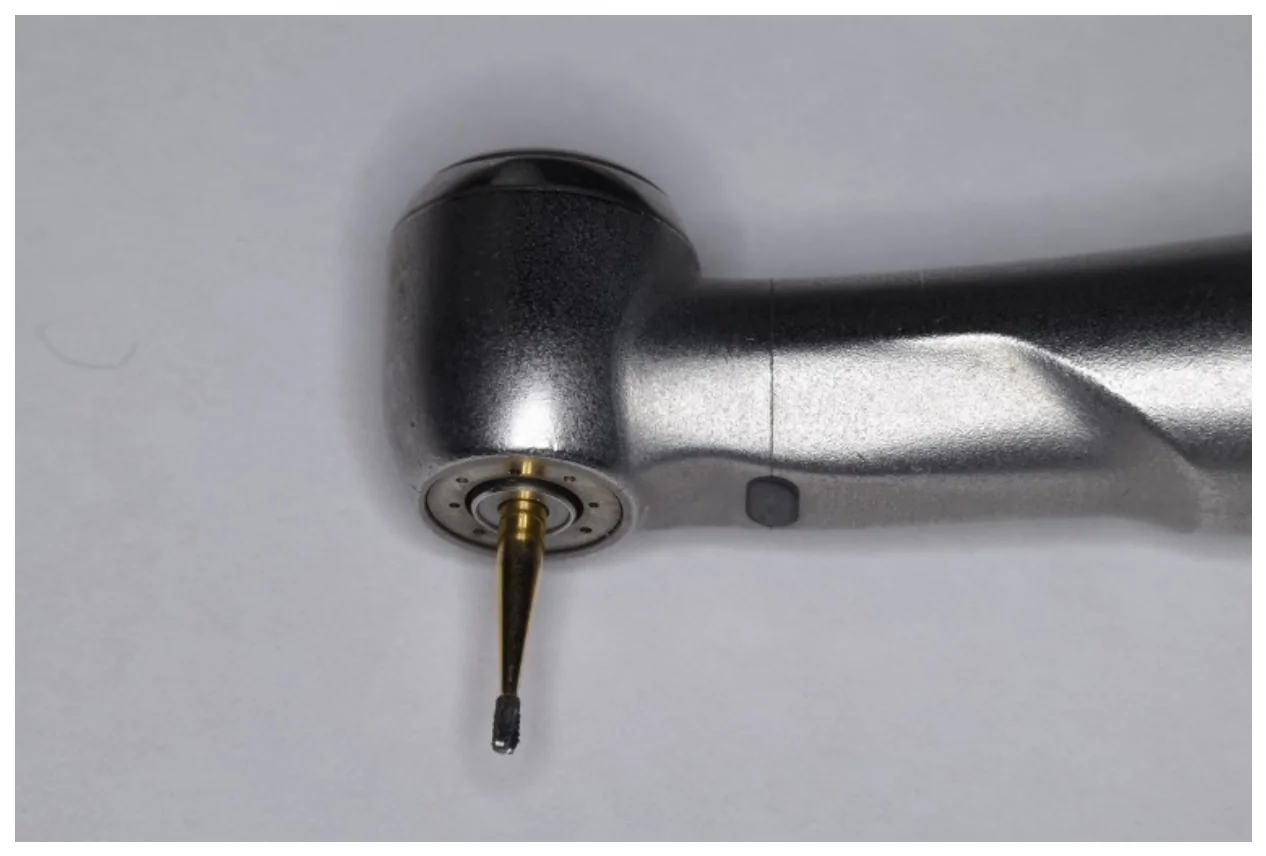
Tungsten carbide bur (FG #1931, head diameter 1.0 mm) used for preparing the slot in the cover screw.

Two weeks later, a revision procedure was performed. Following flap reflection at site #46, a two-step retrieval technique was employed. First, a tungsten carbide bur (Bur Plus Gold #1931, FG shank, head diameter 1.0 mm; Meinan Dental Trading Co., Ltd., Nagoya, Japan) (Figure 2) was used to carefully create a straight-slot notch across the centre of the damaged screw head. The bur was mounted in an air-turbine handpiece (Green Impulse Z-ML; GC Corporation, Tokyo, Japan) operated at the dental unit’s high drive-air setting under continuous water irrigation, with a total cutting time of about one to two minutes. Because the rotational speed of an air-driven turbine is governed by the drive-air pressure and falls substantially under cutting load, the speed during preparation was not measured. The bur was confined to the head of the cover screw and direct contact with the implant body was avoided. The cover screw was made of commercially pure titanium grade 4, which has a lower tensile strength than the titanium–zirconium alloy of the implant body; together with the small volume of the screw head, this allowed the slot to be prepared efficiently. The head of the cover screw measured 3.2 mm in diameter. The slot was extended across its full width, giving a slot approximately 3.2 mm long, and the width of the slot corresponded to the 1.0 mm head diameter of the bur. The depth of the slot was not measured and is estimated to have been of the order of 1 mm.

**Figure 3:**
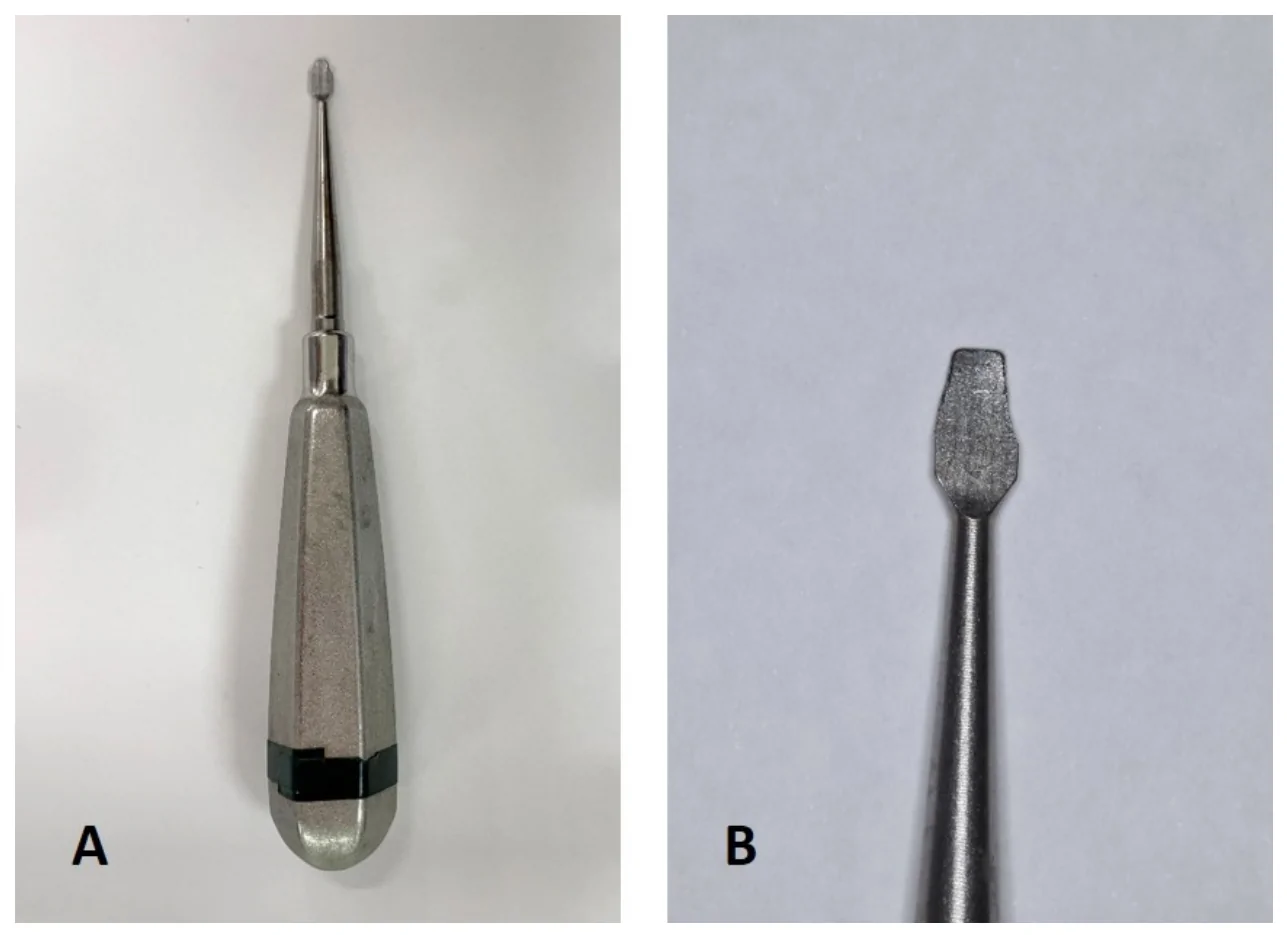
Modified crown remover. (**A**) Overall view showing the large-diameter handle. (**B**) Close-up of the narrowed flat-blade tip.

**Figure 4:**
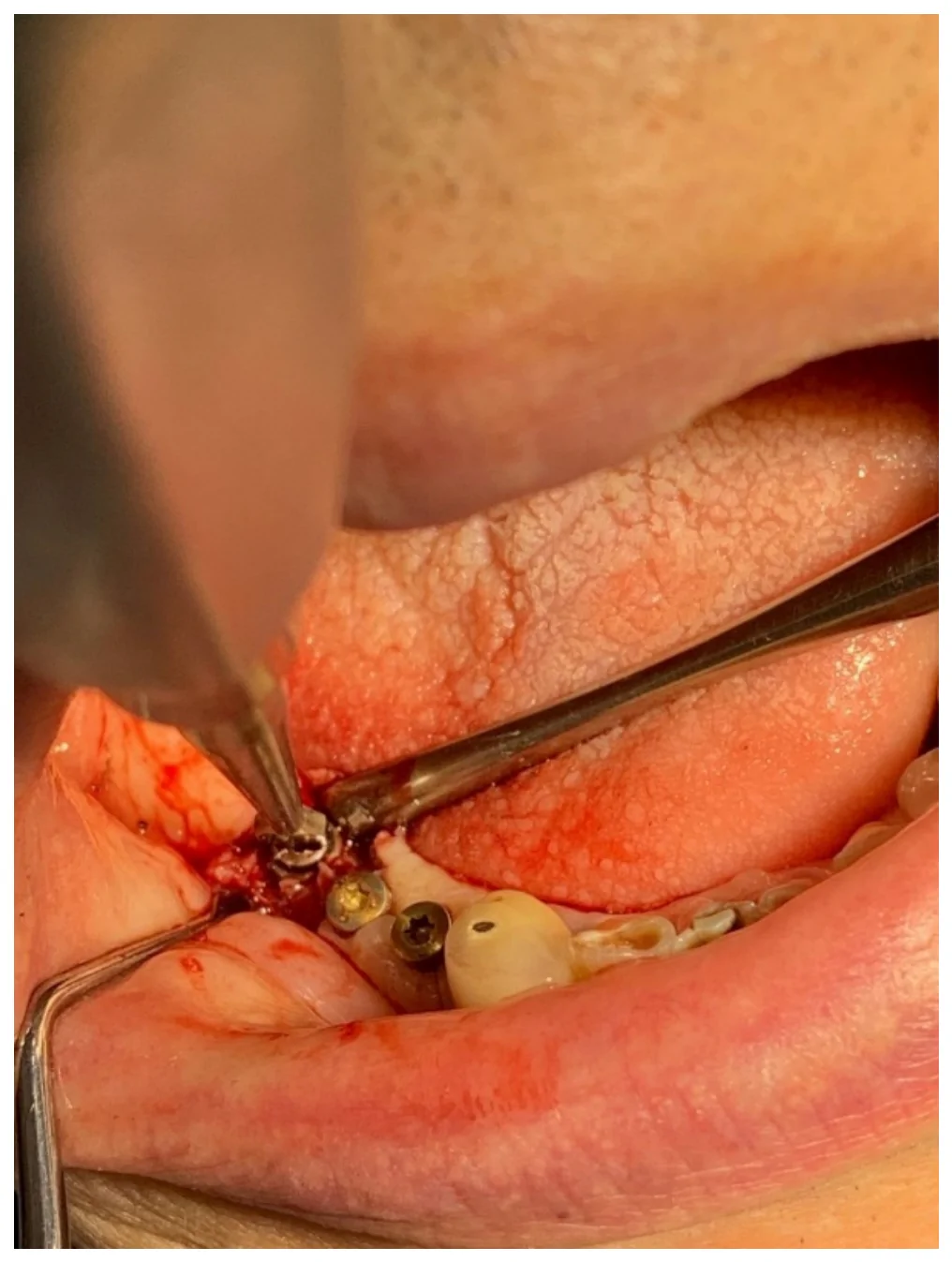
The operator using the modified driver. Torque is applied along the long axis of the cover screw.

**Figure 5:**
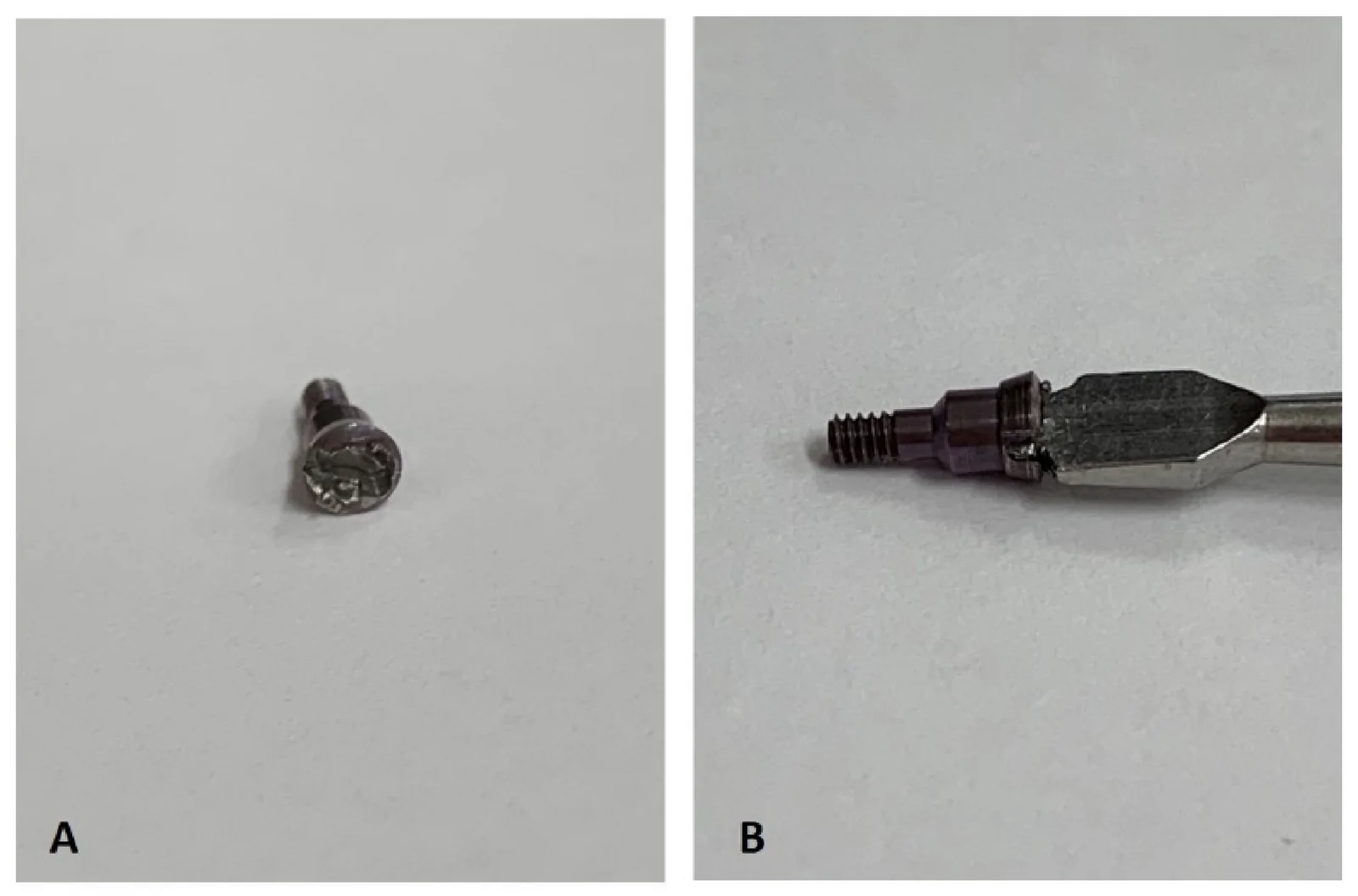
The retrieved cover screw. (**A**) The removed cover screw showing the flat-head-shaped notch cut into its head. (**B**) Alignment between the tip of the modified driver and the notch in the screw head.

**Figure 6:**
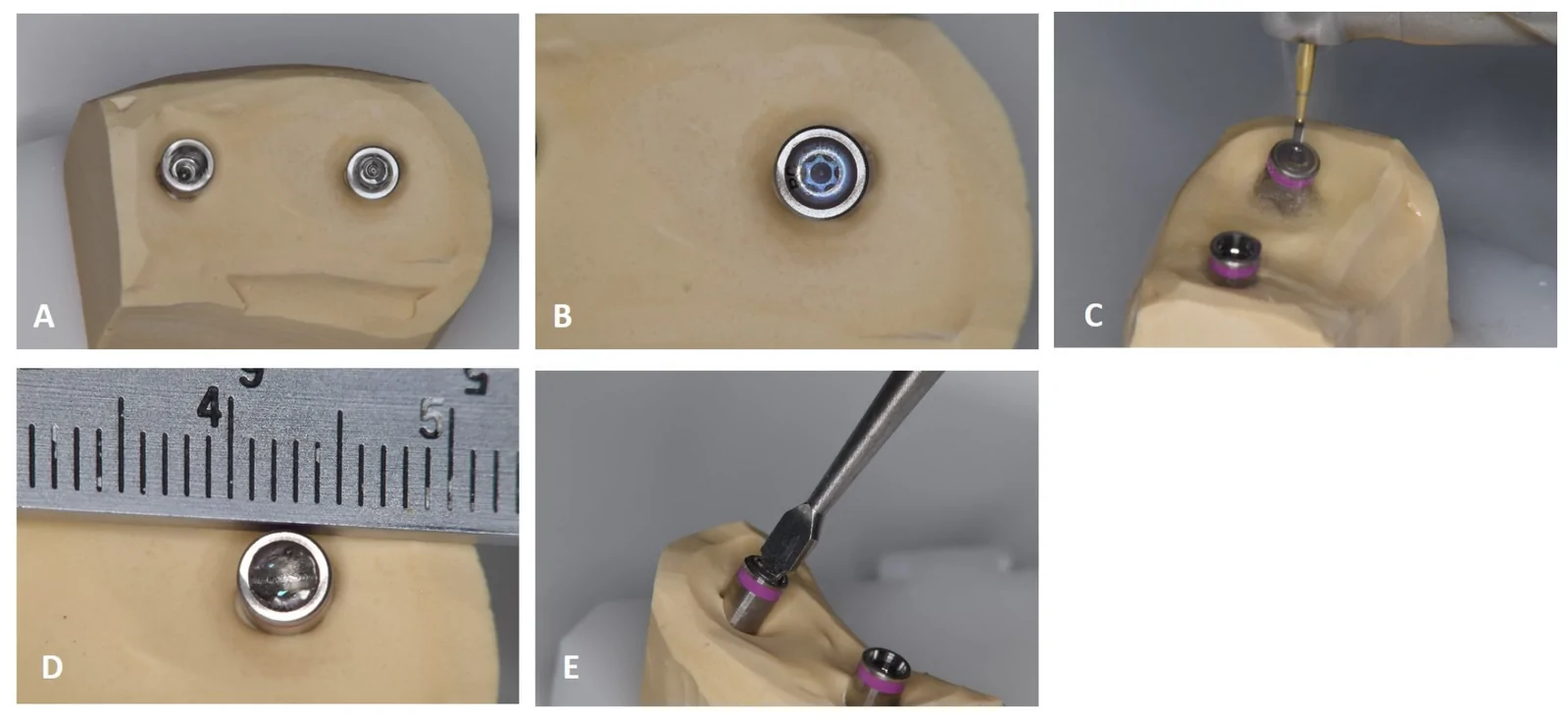
Laboratory demonstration of the slot-preparation and retrieval technique, performed on a bench model with implant analogues after completion of the clinical treatment. (**A**) Two implant analogues seated in the model. (**B**) Occlusal view of a cover screw before slot preparation, showing the original SCS drive slot. (**C**) Preparation of a straight slot across the screw head with a tungsten carbide bur (1.0 mm head diameter) under continuous water irrigation from the air-turbine handpiece. (**D**) The prepared slot, with a millimetre scale positioned for reference. (**E**) The modified driver engaged within the outline of the screw head; the tip was narrowed so that it did not extend beyond the diameter of the cover screw. The demonstration was performed with implant analogues of the same connection type as that used at the treated site. These photographs illustrate the technique and do not depict the clinical case.

In the second step, a modified crown remover with a narrowed flat-blade tip (Removing Driver; YDM Corporation, Tokyo, Japan) was utilized (Figure 3A,B). The working tip was reduced from 4.0 mm to 3.0 mm in width with a carborundum point, so that it engaged the prepared slot while remaining within the 3.2 mm outline of the screw head. This was done because a tip wider than the screw head would transmit removal torque to the implant body itself. Before the procedure, the preparation and engagement were rehearsed on spare cover screws of the same diameter mounted in autopolymerising resin. Upon engaging the tool and applying manual counter-clockwise pressure, the cover screw turned with a distinct sensation of release and was removed (Figure 4). The torque required was not measured. A distinct slot was visible on the retrieved screw head (Figure 5A,B). A healing abutment was then seated at the specified torque, and the flap was sutured. Metallic debris generated during cutting was flushed from the field by the water spray of the handpiece and by saline irrigation at the end of the procedure. No gauze or barrier isolation was used. The preparation and engagement of the modified driver are illustrated on a bench model in Figure 6.

### 2.5. Postoperative Course and Provisional Restoration

**Figure 7:**
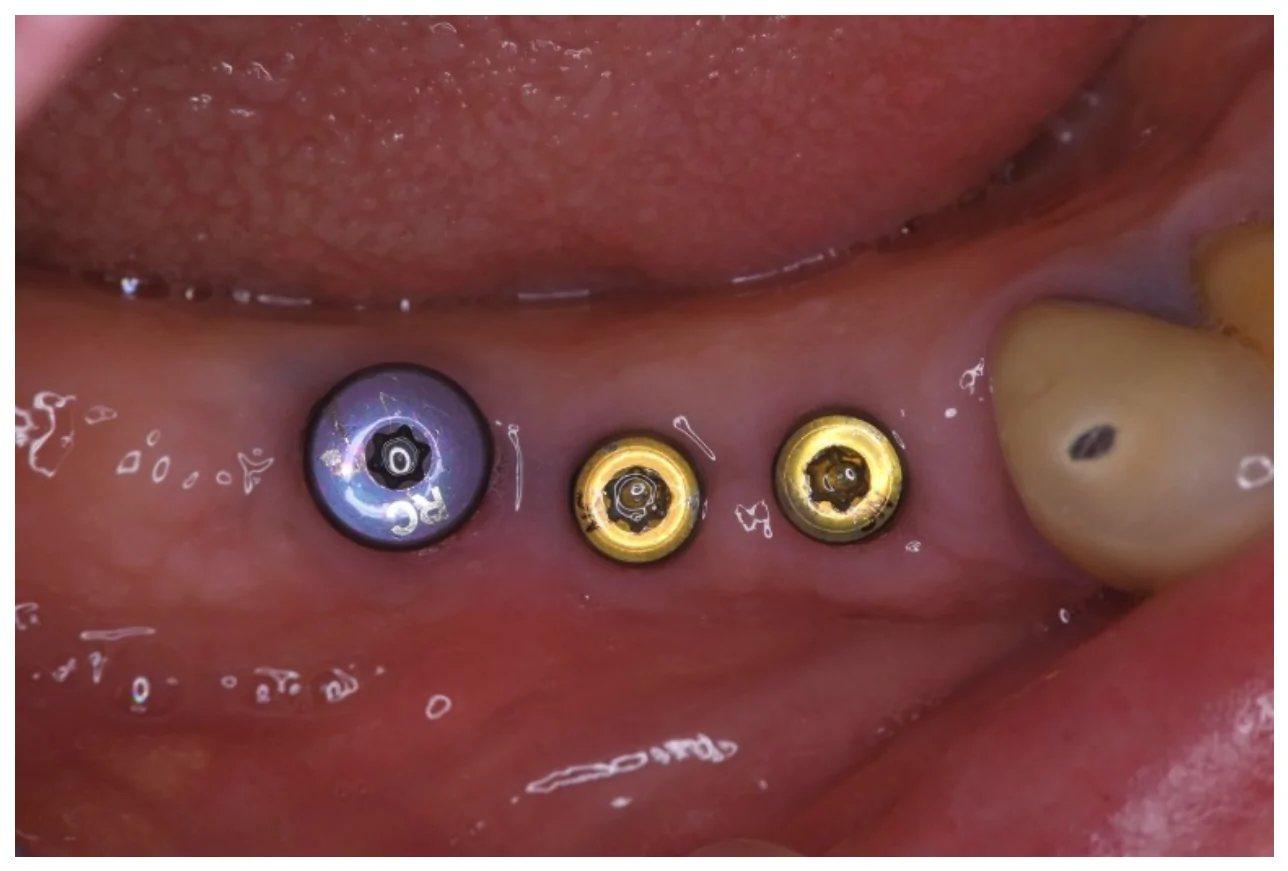
Occlusal view following wound healing after the revision surgery. No signs of inflammation are observed in the surrounding soft tissues.

**Figure 8:**
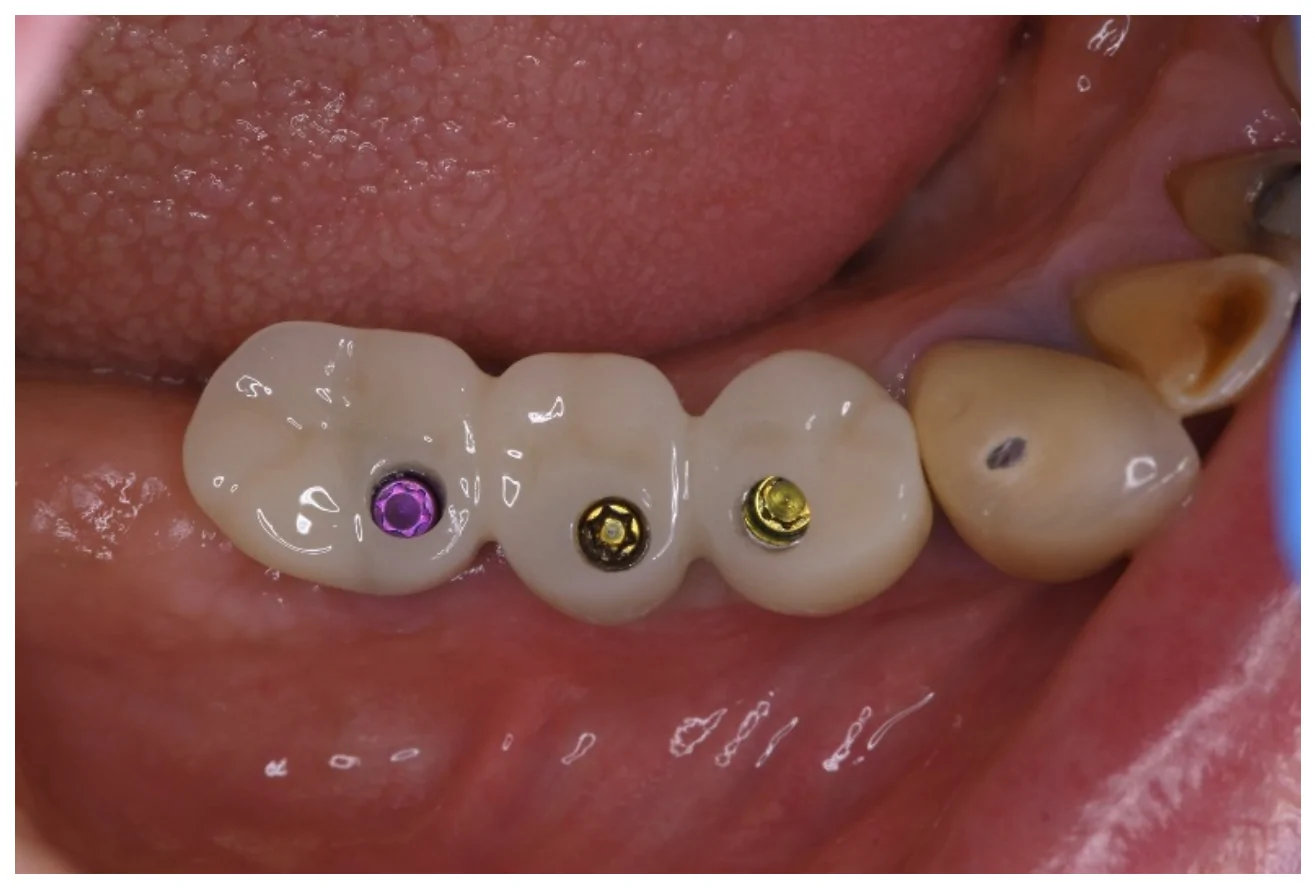
Acrylic resin provisional crown in place.

Postoperative healing was uneventful, with no signs of infection, persistent pain, or neurological deficit (Figure 7). Following soft tissue maturation, an acrylic resin provisional crown was fabricated and delivered (Figure 8).

### 2.6. Definitive Restoration and Follow-Up

**Figure 9:**
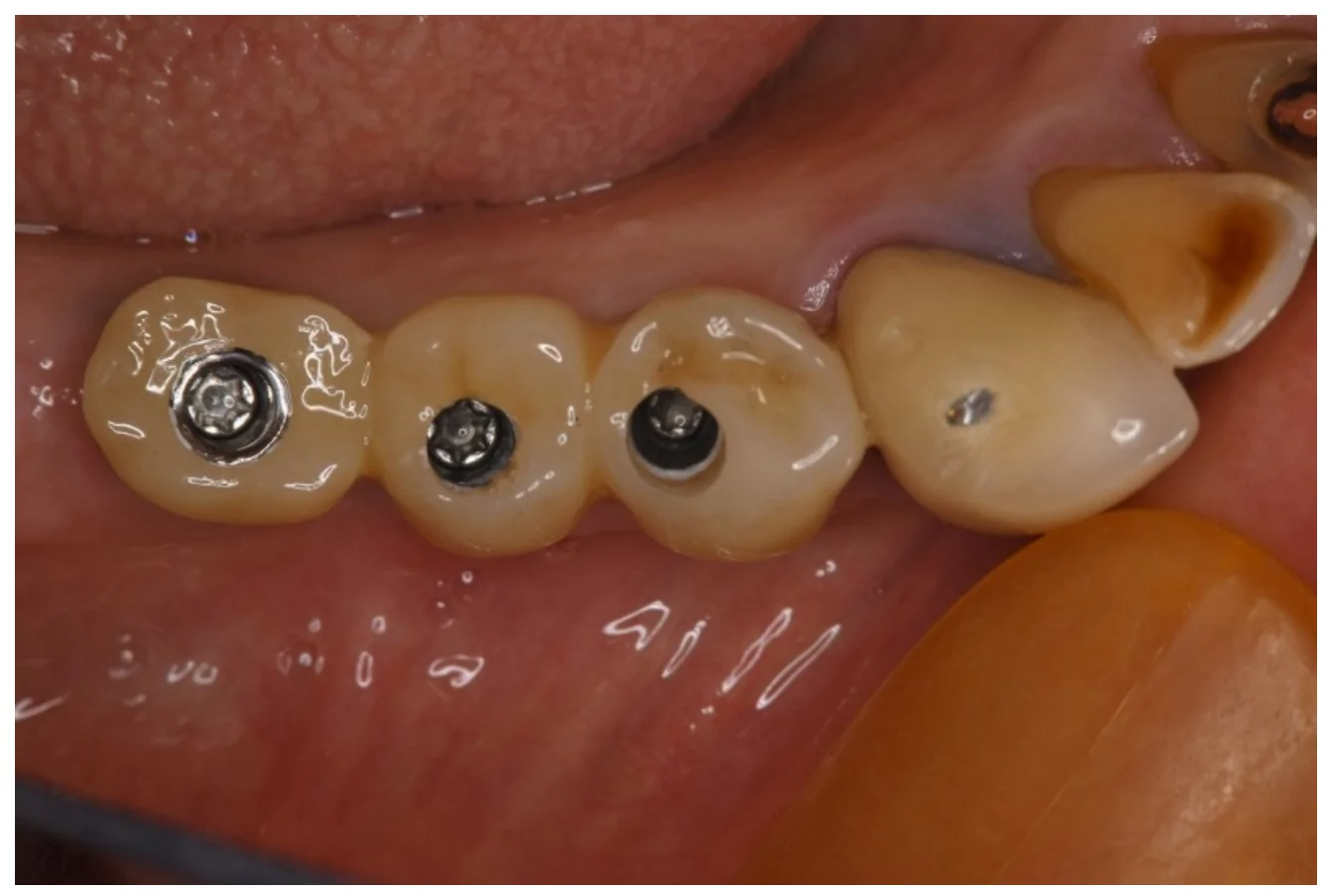
Occlusal view of the definitive screw-retained, splinted zirconia fixed partial denture spanning #44–#46, delivered on 8 June 2026.

After a provisional period of approximately one month, during which peri-implant soft tissue contours were confirmed to be stable, the definitive treatment plan was finalized. A splinted zirconia fixed partial denture spanning #44–#46 was fabricated and delivered on 8 June 2026 using a screw-retained design (Figure 9). The definitive abutment engaged the internal threads of the #46 implant body without resistance and seated at the manufacturer-recommended torque of 35 Ncm, which supports preservation of the functional integrity of the internal connection. Microscopic damage cannot be excluded on clinical grounds.

**Figure 10:**
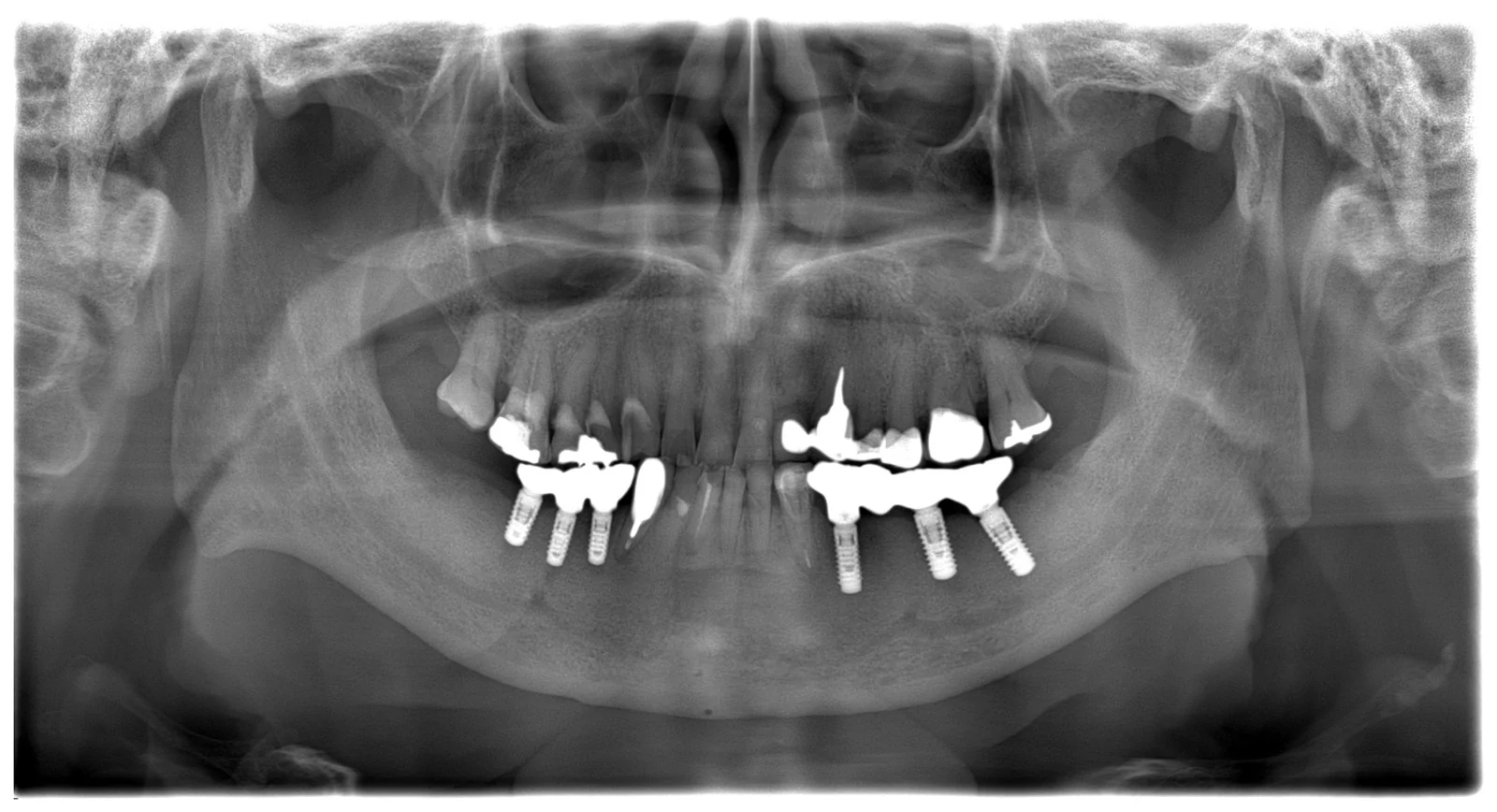
Panoramic radiograph obtained on the day of delivery of the definitive prosthesis (8 June 2026). The reimplanted contralateral #36 site is also visible, without abnormal findings.

**Figure 11:**
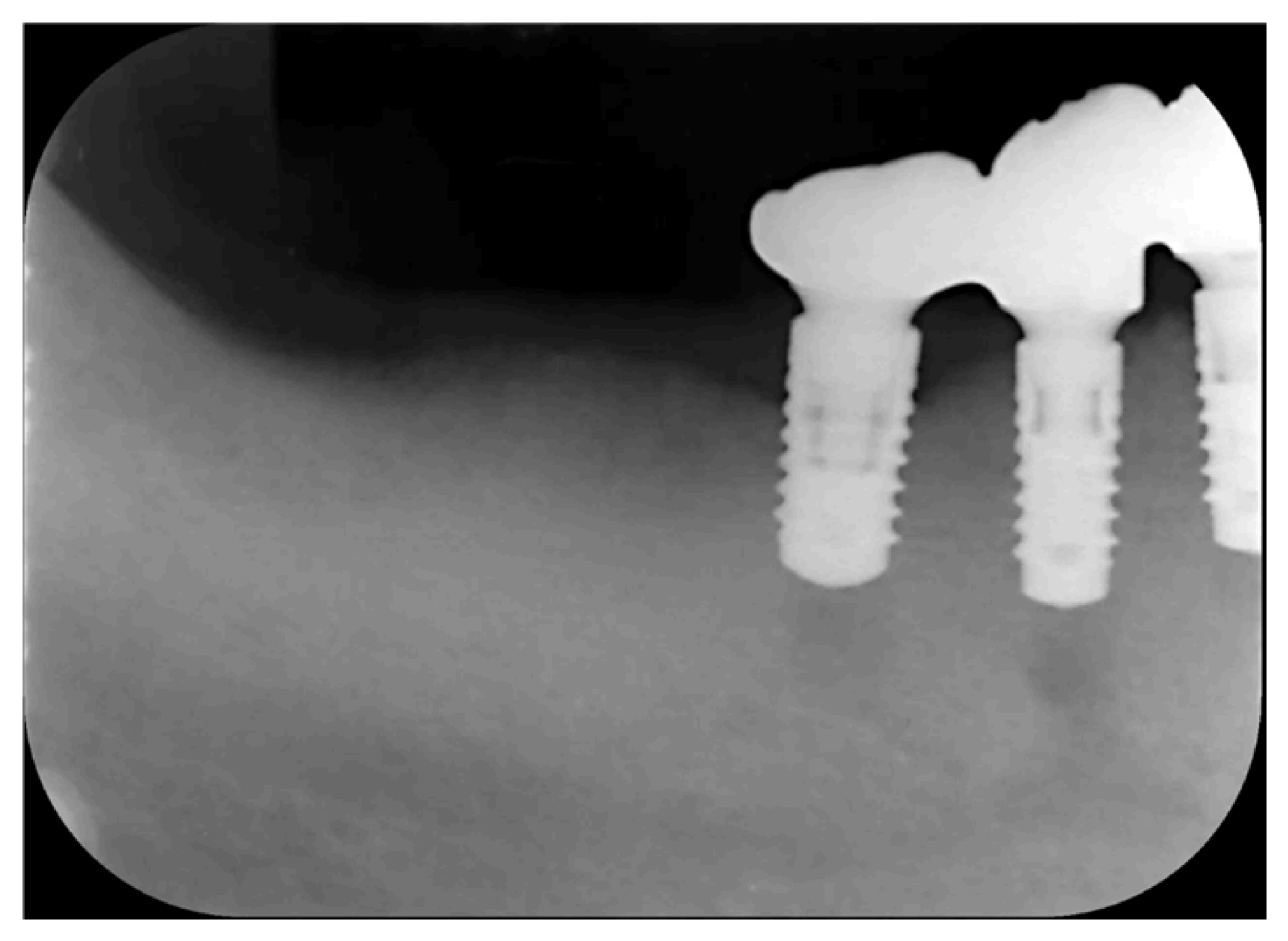
Periapical radiograph of the right posterior region obtained seven weeks after delivery of the definitive prosthesis (29 July 2026). The distance from the implant platform to the crestal bone was greater at #46 than at #44 and #45.

A panoramic radiograph was obtained on the day of delivery (Figure 10). A periapical radiograph of the right posterior region was obtained seven weeks later, on 29 July 2026 (Figure 11). At the most recent review, on 24 August 2026, the patient reported normal masticatory function without discomfort, and no screw loosening or peri-implant mucositis was observed. Longer-term follow-up will be required to confirm the durability of this outcome.

## 3. Discussion

### 3.1. Possible Mechanisms of Fixation

Cold welding is one possible explanation for the fixation observed in this case. This phenomenon occurs when the surface oxide layer is disrupted at the interface between two contacting metal surfaces, allowing direct metal-to-metal contact and adhesion at the atomic level. Cold welding has been reported as a cause of complications in implant components [5,6] and is well documented in orthopaedic fracture fixation, where a recent systematic review identified it in 58 of 1654 screws (3.5%) [10].

The two components in this case were not made of the same alloy. The implant body was a titanium–zirconium alloy, while the cover screw was commercially pure titanium grade 4. Fixation occurred nonetheless. This suggests that dissimilarity of the bulk alloy does not in itself prevent seizure, presumably because both surfaces carry a titanium dioxide passive layer and the relevant interaction takes place at that oxide interface rather than between the bulk alloys.

The difference in mechanical properties between the two components may also account for the pattern of damage observed. Commercially pure titanium grade 4 has a lower tensile strength than the titanium–zirconium alloy of the implant body, for which the manufacturer specifies a minimum of 850 MPa. As torque was applied, the softer cover screw may therefore have yielded first, so that its drive slot deformed while the internal connection of the implant remained intact. This interpretation is consistent with the uneventful seating of the definitive abutment, although it could not be verified directly.

Contamination of the interface by blood or saliva at the time of placement offers another explanation. Óvári et al. list blood and debris between the implant threads and the screw among the biological causes of cover screw failure [5]. The cover screw was placed under a reflected flap, so contact with blood and with crevicular fluid during the four-month submerged period cannot be excluded, and corrosion or the accumulation of organic deposits at the interface may have contributed. The interface was not examined after retrieval, so this possibility could neither be confirmed nor ruled out.

**Figure 12:**
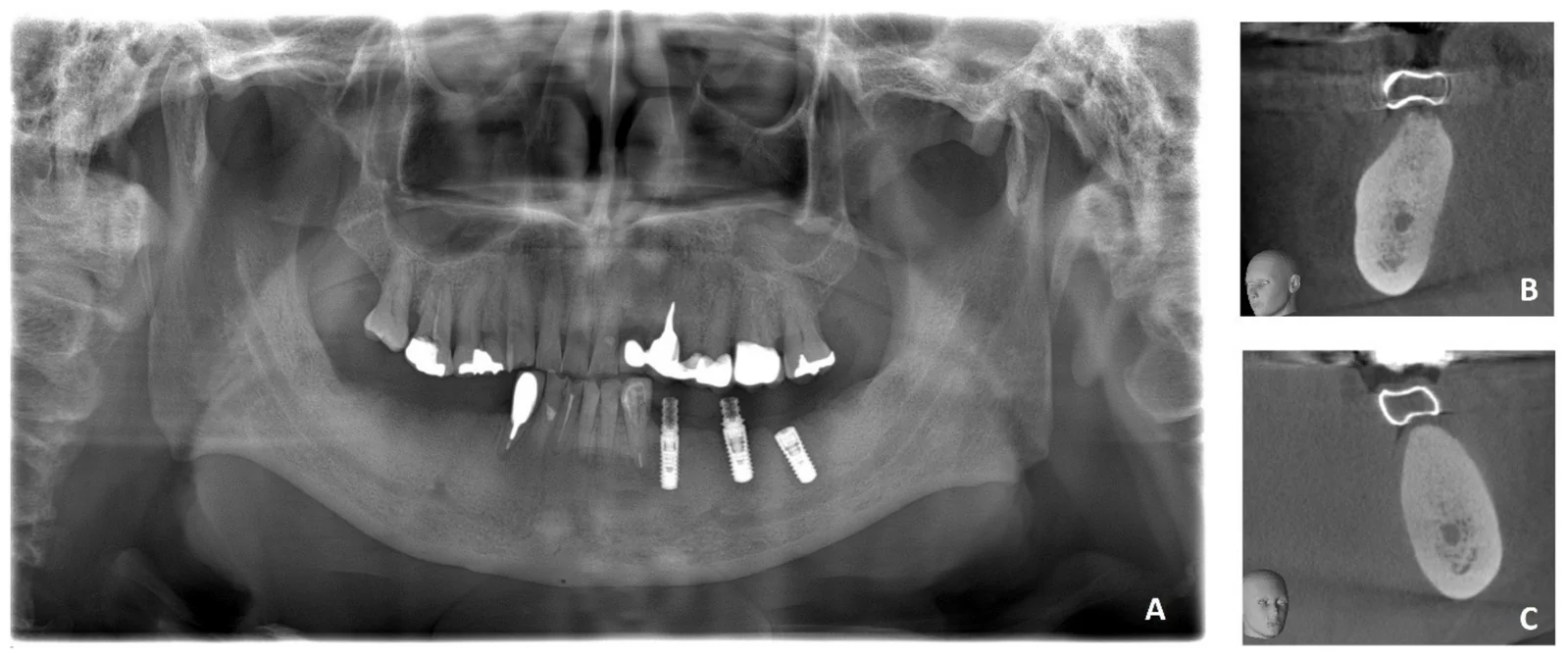
Preoperative radiographic findings. (**A**) Panoramic radiograph showing a diffuse increase in radiopacity throughout the mandible. (**B**) Coronal CBCT sections at the right (#44–#46) and (**C**) left (#36) planned implant sites, obtained with a radiographic stent in place, showing visually homogeneous, radiopaque cortical bone bilaterally, corresponding to Lekholm and Zarb Type 1 bone quality [13].

**Figure 13:**
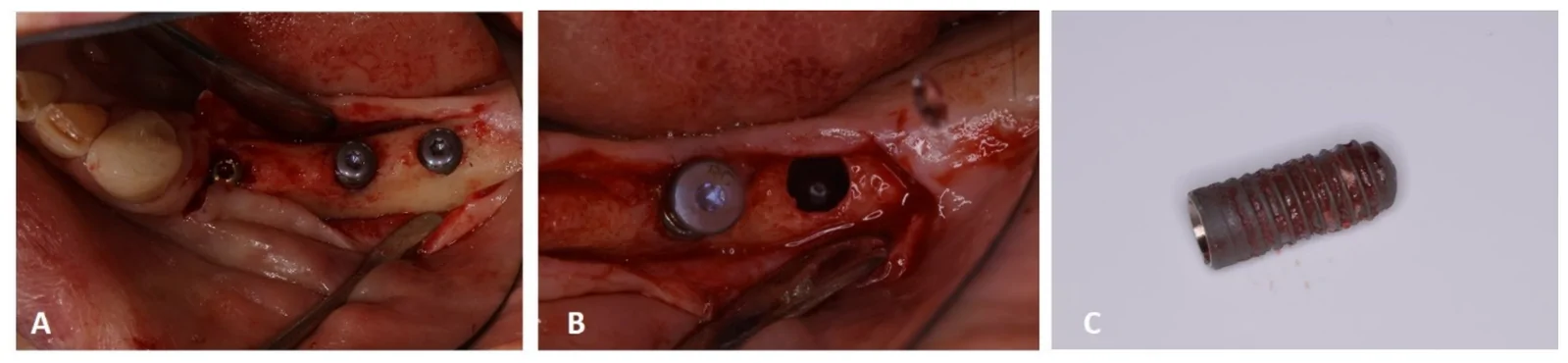
Treatment course of the #36 implant on the contralateral side. (**A**) Primary stability achieved at placement. (**B**) Loss of osseointegration observed at second-stage surgery. (**C**) The retrieved implant body.

A further feature of this case was a diffuse increase in the radiopacity of the mandible on the preoperative panoramic radiograph (Figure 12A). During the initial implant placement on the contralateral side (#36), bone preparation was notably difficult owing to extreme bone density, which ultimately led to failure of osseointegration and required reimplantation (Figure 13). Preoperative CBCT similarly demonstrated visually homogeneous, radiopaque cortical bone bilaterally (Figure 12B,C), so the appearance was not an artifact of panoramic projection. Bilateral Type 1 bone of this uniformity is uncommon in routine implant practice. In bone of very high density, insertion torque may increase unintentionally, which could subject the interface between the cover screw and the internal structure of the implant to compressive stress; remodelling within dense bone might also exert pressure on the implant body during healing. Both possibilities are speculative, and the relationship between high bone density and cover screw fixation has received little attention in the literature.

### 3.2. Retrieval Technique and Comparison with Reported Methods

There is a paucity of systematic reports regarding the management of immobile cover screws, and no standardized protocol has been established [8]. While common prosthetic complications such as screw fractures and abutment damage are well documented [14,15], case reports specifically addressing seized cover screws or stripped drive slots are rare. In this case, the stripping of the screw head during the attempt with the torque driver was likely due to stress concentration at the driving surfaces of the Screw Carrying System (SCS) [16]. The rotational resistance caused by the fixation would have focused the applied torque onto a small cross-sectional area of the drive slot, leading to plastic deformation.

The retrieval technique can be explained by two mechanical factors. First, the modified crown remover featured a handle with a larger diameter. This increased the moment arm, allowing for more efficient transmission of removal torque with the same manual force [17]. Second, the contact area between the prepared straight slot and the flat-blade driver is larger than the point–contact interface of the original SCS design. This increased surface area reduced the stress concentration per unit area, allowing the screw to be loosened without further damage.

The technique differs from the method reported by Óvári et al., in which a cold-welded cover screw with a worn drive slot was retrieved by drilling through the centre of the screw with a tapered carbide bur from a manufacturer-specific retrieval kit [5]. That approach sacrifices the screw and introduces instrumentation into the internal channel of the implant, and the authors advised against its use where bone ingrowth may be present. In the present case the slot was prepared on the external surface of the screw head only, the screw was retrieved intact, and no instrument entered the internal channel. The instruments used were not specific to the implant system.

### 3.3. Verification of Internal Connection Integrity

A principal concern associated with any mechanical retrieval technique is iatrogenic damage to the internal threads of the implant body, which cannot be reliably excluded on the basis of intraoperative visual inspection alone. In the present case the definitive abutment engaged the internal threads without resistance, achieved the manufacturer-recommended seating torque, and has since functioned under occlusal load without screw loosening or prosthetic complication. This supports preservation of the functional integrity of the connection, although microscopic damage cannot be excluded on clinical grounds. Functional assessment of this kind is a useful endpoint when reporting salvage techniques, since a retrieval that preserves the implant body but compromises the internal connection would ultimately fail to avert implant loss.

### 3.4. Practical Considerations

Several findings were available before the second-stage surgery: a diffuse increase in mandibular radiopacity on both panoramic radiography and CBCT, Type 1 bone quality with unusually high resistance during osteotomy preparation, and a documented history of osseointegration failure at the contralateral site. Whether these constitute a risk profile for cover screw seizure cannot be determined from a single case.

The cover screws in this case were hand-tightened with a manual screwdriver, without a torque wrench, yet seizure still occurred. Excessive initial tightening torque alone therefore does not account for the complication, and hand-tightening should not be assumed to eliminate this risk. Whether cold welding or bone-mediated compressive forces contributed cannot be established here.

If a cover screw does not yield to a manual screwdriver, escalating torque through a contra-angle driver carries a risk of converting a retrievable screw into a stripped one, as occurred here. In this case the driver was used with torque attachments of 20 Ncm and 35 Ncm, the latter corresponding to the tightening torque recommended for abutments in this system, and no intermediate value was available. In retrospect, deformation of the drive slot may already have begun at 20 Ncm. Óvári et al. reported stripping of a cover screw drive slot with a reverse torque that did not exceed 30 Ncm [5]. These two observations suggest that a drive slot can be damaged at or below the recommended tightening torque, and that suspending the procedure is likely to be the lower-risk course.

A slotted driver mounted on a torque wrench would allow the removal torque to be controlled, but no such adapter was available for this system. A seized screw also releases suddenly once it begins to turn, and manual control was preferred so that the release could be felt and the movement stopped. Whether a torque-controlled slotted driver would be safer has not been tested and would be worth examining.

### 3.5. Peri-Implant Bone Levels and Possible Local Factors at Site #46

Peri-implant marginal bone levels were assessed on the digital periapical radiograph obtained seven weeks after delivery of the definitive prosthesis (Figure 11). The radiograph was taken using the paralleling technique, and measurements were performed on a chairside monitor with the digital ruler function of a radiograph viewing system (TechM@trix, SDS Viewer; TechMatrix Corporation, Tokyo, Japan) by a single examiner (K.S.), adapting the method previously reported by our group [18]. The vertical distance from the mesial and distal margins of the implant platform to the crestal bone level was 0 mm and 0.7 mm at #44, 0 mm and 0.8 mm at #45, and 2.2 mm and 2.8 mm at #46. As these were bone-level implants placed with the platform at the level of the crest, these distances approximate the extent of crestal bone remodelling. No standardised baseline periapical radiograph was available, so the absolute change over time could not be calculated.

The bone level at #46 was lower than at the two adjacent implants, which had been placed in the same patient during the same operation and restored with the same splinted prosthesis. Several local factors may have contributed, and they cannot be distinguished from one another in a single case.

First, site #46 underwent two additional flap elevations within a two-week interval, at the second-stage surgery and at the revision procedure, whereas #44 and #45 were exposed only once.

Second, heat generated during notch preparation may have been conducted to the crestal bone. Although the bur was confined to the head of the cover screw and direct contact with the implant body was avoided, the cover screw is threaded into the implant platform, and titanium conducts heat readily. Neither the rotational speed of the air-turbine handpiece nor the temperature at the implant–bone interface was measured.

Third, metallic debris generated during cutting was flushed from the field by the water spray and by saline irrigation, but no gauze or barrier isolation was used, and retention of small particles within the peri-implant tissues cannot be excluded.

Fourth, the patient is totally blind and his plaque control was assessed as suboptimal. Site #46 is the most posterior of the three implants and carries the largest-diameter fixture, making it the least accessible to self-performed cleaning.

These observations indicate that although the implant body was preserved, the retrieval procedure may have been accompanied by a loss of crestal bone at the treated site. This possibility should be weighed when the technique is considered.

### 3.6. Differential Considerations for the Radiographic Appearance of the Mandible

The mandible showed a diffuse increase in radiopacity on both panoramic radiography and CBCT, and Type 1 bone was encountered bilaterally. The term osteosclerosis has been avoided here because the differential diagnosis was not pursued during clinical care. Paget disease of bone, osteopetrosis, renal osteodystrophy, osteoblastic metastasis and chronic diffuse sclerosing osteomyelitis were not formally excluded, and no serum alkaline phosphatase, calcium or phosphate values were available for review.

The patient had received oral prednisolone between the ages of 20 and 25. Long-term corticosteroid exposure reduces rather than increases bone density, and the exposure ended more than forty years before presentation, so it is unlikely to account for the radiographic appearance. He had never received a bisphosphonate or denosumab, and had received neither radiotherapy nor chemotherapy for the resected colorectal cancer, so a drug-related or radiation-related cause can reasonably be excluded. He reported a gradual decline in renal function, and renal osteodystrophy therefore cannot be excluded, although no laboratory data were available to assess this. He has been taking an antihypertensive agent for five years; an influence of antihypertensive medication on peri-implant parameters has been reported [18], but a relationship with mandibular bone density has not been established.

### 3.7. Limitations

The cause of fixation was not verified by surface or metallurgical analysis, so cold welding, contamination of the interface by biological fluids, and the influence of bone density all remain unverified.

The rotational speed of the air-turbine handpiece and the temperature generated at the implant–bone interface were not measured. An air-driven turbine exhausts air into the operative field, and in an open surgical wound this carries a risk of subcutaneous emphysema. An electric handpiece or a surgical motor without air exhaust would be preferable.

Metallic debris was flushed by the water spray and by saline irrigation, but no gauze or barrier isolation was used, and retention of particles in the peri-implant tissues cannot be excluded. Isolation of the field would be advisable.

The dimensions of the prepared slot were recorded on a bench model rather than at the time of surgery, and the modified driver has not been standardised.

Peri-implant bone levels were assessed on a single periapical radiograph. No standardised baseline radiograph was available, so the change over time could not be calculated, and the measurements approximate rather than quantify crestal bone remodelling.

The definitive prosthesis has been in function for a short period only, and longer follow-up is required.

The patient has a history of Behçet’s disease, hyperuricemia, hypertension and resected colorectal cancer, and reports a gradual decline in renal function. No laboratory data were available for review, so a metabolic contribution to the radiographic appearance of the mandible could not be assessed.

## 4. Patient Perspective

The following reflects the patient’s own statements as documented in the clinical record (Subjective findings, SOAP notes) at the relevant time points. Immediately after the failed cover screw retrieval attempt at second-stage surgery, when he was informed that a further surgical procedure would be required, the patient reported feeling anxious about undergoing an additional operation. At the time of delivery of the definitive prosthesis, he described the outcome as comfortable, with restored chewing function and no ongoing concerns.

## 5. Conclusions

This report describes the successful use of a stepwise technique for retrieving a seized implant cover screw with a stripped drive slot. A straight slot was prepared on the screw head with a tungsten carbide bur and engaged with a modified crown remover, and the screw was retrieved intact without instrumentation within the internal channel of the implant. The definitive prosthesis was delivered and remained in function at the most recent review. At the treated site, however, the distance from the implant platform to the crestal bone was greater than at the two adjacent implants placed in the same operation, and the contribution of the additional surgery, of heat generated during cutting, of metallic debris, and of the patient’s limited capacity for plaque control cannot be separated.

Cover screw seizure is uncommon, but it may lead to loss of the entire implant if managed inappropriately. The present case indicates that a drive slot can be damaged at or below the tightening torque recommended for the system, and that escalating torque when a cover screw fails to yield is best avoided. Whether the technique described here is reproducible, and whether it can be performed without crestal bone loss, will require further cases.

## Figures and Tables

**Table 1:** Reported techniques for the retrieval of seized or damaged implant components.

Component/Condition	Reported Technique	Advantages	Limitations	Ref.
Fractured abutment screw	Ultrasonic scaler, explorer or Hedström file, manufacturer-specific retrieval kits	Several established options; implant body preserved in most cases	Difficult when the fragment lies deep within the implant; risk of damage to internal threads; kits are system-specific	[9]
Cold-welded prosthetic abutment	Modified hexagonal driver; sectioning of the abutment	Effective where conventional loosening fails	Patient discomfort; heat generation; abutment sacrificed	[6]
Cold-welded fixation screw (orthopaedic)	Conical extraction devices, carbide burs, trephines	Established in orthopaedic practice; incidence quantified (58 of 1654 screws, 3.5%)	Not transferable to the internal geometry of dental implants	[7,10]
Seized cover screw, drive slot intact	Engagement of the retained drive slot with adapted instruments	Non-destructive to the screw	Requires an intact drive slot	[8]
Cold-welded cover screw with worn drive slot	Drilling through the centre of the screw with a tapered carbide bur from a manufacturer-specific retrieval kit	Effective; implant preserved	Screw destroyed; instrumentation enters the internal channel; the authors advise against use where bone ingrowth may be present; kit required	[5]
Present case: seized cover screw with stripped drive slot	Preparation of a new external slot on the screw head with a tungsten carbide bur, followed by engagement with a modified crown remover	Screw retrieved intact; no instrumentation within the internal channel; no system-specific kit required	Single case; slot dimensions not standardised; heat generation and metallic debris not controlled or measured	—

## Data Availability

The original contributions presented in this study are included in the article. Further inquiries can be directed to the corresponding author.

## Abbreviations

The following abbreviations are used in this manuscript:

CARE	CAse REport guidelines
CBCT	Cone-beam computed tomography
FG	Friction grip
NC	Narrow CrossFit
RC	Regular CrossFit
SCS	Screw Carrying System
